# Variation in the operationalisation of dose in implementation of health promotion interventions: insights and recommendations from a scoping review

**DOI:** 10.1186/s13012-019-0899-x

**Published:** 2019-06-06

**Authors:** Samantha Rowbotham, Kathleen Conte, Penelope Hawe

**Affiliations:** 1 Menzies Centre for Health Policy, School of Public Health, Faculty of Medicine and Health, Charles Perkins Centre, University of Sydney, Sydney, Australia; 2 The Australian Prevention Partnership Centre, Sydney, Australia

**Keywords:** Dose, Health promotion, Implementation, Evaluation, Intervention delivery

## Abstract

**Background:**

While ‘dose’ is broadly understood as the ‘amount’ of an intervention, there is considerable variation in how this concept is defined. How we conceptualise, and subsequently measure, the dose of interventions has important implications for understanding how interventions produce their effects and are subsequently resourced and scaled up. This paper aims to explore the degree to which dose is currently understood as a distinct and well-defined implementation concept outside of clinical settings.

**Methods:**

We searched four databases (MEDLINE, PsycINFO, EBM Reviews and Global Health) to identify original research articles published between 2000 and 2015 on health promotion interventions that contained the word ‘dose’ or ‘dosage’ in the title, abstract or keywords. We identified 130 articles meeting inclusion criteria and extracted data on how dose/dosage was defined and operationalised, which we then synthesised to reveal key themes in the use of this concept across health promotion interventions.

**Results:**

Dose was defined in a variety of ways, including in relation to the amount of intervention delivered and/or received, the level of participation in the intervention and, in some instances, the quality of intervention delivery. We also observed some conflation of concepts that are traditionally kept separate (such as fidelity) either as slippage or as part of composite measures (such as ‘intervention dose’).

**Discussion:**

Dose is not a well-defined or consistently applied concept in evaluations of health promotion interventions. While current approaches to conceptualisation and measurement of dose are suitable for interventions in organisational settings, they are less well suited to policies delivered at a population level. Dose often accompanies a traditional monotonic linear view of causality (e.g. dose response) which may or may not fully represent the intervention’s theory of how change is brought about. Finally, we found dose and dosage to be used interchangeably. We recommend a distinction between these terms, with ‘dosage’ having the advantage of capturing change to amount ‘dispensed’ over time (in response to effects achieved). Dosage therefore acknowledges the inevitable dynamic and complexity of implementation.

**Electronic supplementary material:**

The online version of this article (10.1186/s13012-019-0899-x) contains supplementary material, which is available to authorized users.

Contributions to the literature

- Dose is considered to be the ‘amount’ of an intervention and is a key concept in many implementation frameworks
- Until now, there has been little systematic investigation exploring variation (and contradictions) in how dose has been conceived and measured within health promotion interventions
- The review reveals that dose delivered and dose received are conceptually distinct and may achieve health effects through separate pathways, encouraging researchers to measure dose in multiple ways and to use complex and complicated logic models to understand how change takes place
- The terms ‘dose’ and ‘dosage’ are often used interchangeably. We explain why they should be kept distinct

## Introduction

Implementation of effective interventions at scale is essential to improving population health [1]. Health promotion interventions often seek to address multiple risk factors simultaneously and at various levels, including the individual, interpersonal, organisational and/or environmental level. Such interventions are often implemented within complex systems, which may respond in unpredictable ways to the intervention [2]. The need to understand how such interventions are implemented is pressing, particularly if we are to draw conclusions about their effectiveness and enable implementation of the same interventions in different settings.

Several frameworks have been developed to guide the evaluation of implementation efforts [3–8]. A key concept in many of these frameworks is *dose*, broadly understood to refer to the ‘amount’ of intervention provided. Dose is a particularly important concept, as understanding how much of an intervention is delivered (and to whom) is critical if we are to replicate and scale up interventions, provide appropriate resourcing and have confidence that observed effects (or lack thereof) can be attributed to the intervention [9]. However, understanding dose in relation to health promotion interventions is not necessarily straightforward.

When it comes to the practice of medicine, dose is commonly understood to mean the amount of a treatment, usually a drug, delivered with the aim of achieving a particular physiological response [10]. While the therapeutic effect of the treatment may vary as a result of other factors (e.g. interactions with other medications, differences in metabolism), the measurement of the dose (i.e. amount) of a drug being delivered is usually straightforward and is typically measured in metric mass units (e.g. milligrammes). However, outside of medicine, the operationalisation and measurement of dose becomes more difficult.

How dose is conceptualised differs somewhat across implementation frameworks. For example, Dusenbury and colleagues [4] define dose as the ‘amount of program content *received* by participants’ (p. 241, italics added), while Dane and Schneider [3] refer to ‘exposure,’ which focuses on the *delivery* of the intervention and includes ‘(a) the number of sessions implemented; (b) the length of each session; or (c) the frequency with which program techniques were implemented’ (p. 45). Steckler and Linnan [11] distinguish between ‘dose delivered’, defined as the ‘number or amount of intended units of each intervention or each component delivered or provided,’ and ‘dose received,’ which is ‘the extent to which participants actively engage with, interact with, are receptive to, and/or use materials or recommended resources’ (p. 12). In some of these frameworks, dose is a single element of a larger framework. In others, dose is a composite concept made up of multiple elements. For example, Wasik and colleagues [12] distinguish between implementation and intervention dose and further subdivide the latter into the amount of intervention intended, offered, and received. Legrand and colleagues [7] provide an equation to calculate dose that involves measuring delivery quantity and quality and participation quantity and quality. Finally, Cheadle and colleagues [13] introduce the concept of ‘population dose’ which is defined as a product of the reach of an intervention and the strength of the effect (estimated as the effect on each person reached by the intervention). See Table 1 for an overview of how dose is conceptualised across these frameworks.

**Table 1:** Overview of selected theoretical frameworks that include the concept of dose

Article	Terms used	Definition
Cheadle et al. [13]	Population dose	Dose is defined as a product of intervention reach (number of people ‘touched’ by the intervention) × strength (estimated effect of intervention on each person). Dose = reach × strength
Collins et al. [14]	Dosage	Defines dose as the amount of intervention to be delivered, tailored to each individual in order to achieve the desired response (i.e. dose is a function of the efforts of intervention providers)
Dane & Schneider [3]	Dosage	Dosage is one of eight components of ‘implementation’ and is defined as how much of the programme has been delivered, and includes quantity and intervention strength (i.e. dose is a function of the efforts of intervention providers)
Legrand et al. [7]	Dose	Dose is defined as a product of the following: Delivery quantity (DQt) Delivery quality (DQl) Participation quantity (PQt) Participation quality (PQl) Dose = DQt × (mean DQl, PQt, PQl/mas) (where mas = common maximal assignable score) (i.e. dose is a function of the efforts of intervention providers and participants)
Steckler & Linnan [11]	Dose delivered	Dose is identified as one component within a process evaluation framework and is defined as the number/amount of intended units delivered/provided (i.e. dose is a function of the efforts of intervention providers)
	Dose received	Extent to which participants engage or interact with are receptive or use intervention (i.e. dose is a function of the efforts of intervention participants)
Wasik et al. [12]	Implementation dosage	Activities necessary to for intervention to be carried out with fidelity, including dosage of training received by those who will deliver the intervention (e.g. amount of time instructors spend training intervention providers)
	Intervention dosage	Amount of intervention, which includes the *dosage intended* for the programme model, *dosage offered* by the service provider and *dosage received* by the intervention recipient
	Cumulative dosage	The amount of intervention a participant receives over the life of a programme, where Cumulative dosage = session duration × frequency × intervention duration or length of programme enrolment

How we conceptualise and measure the dose of interventions has important implications for understanding how interventions produce their effects, and for whether and how interventions are resourced and scaled up. Policy makers want to know what they need to fund and implement to produce population-level health gains, while practitioners want to know ‘how much’ they need to do to ensure that health promotion programmes ‘work’ at a local level. As indicated in Table 1, there is considerable variation in how dose is conceptualised and measured. These incongruences are important regardless of whether one subscribes to the view that the fidelity of the intervention lies in the faithful replication of particular core programme components [15] or in more complex understanding of the intervention where effects are seen to derive more from interaction with the dynamic properties of the system into which the intervention is introduced [16, 17]. It is therefore vital to think critically about what dose means so that researchers and policymakers can gain the evidence they need to inform decisions.

### Aim

The variation in how dose is defined across the frameworks in Table 1 prompted us to undertake an investigation of the nature and extent of differences in how dose has been conceptualised and measured in the implementation of health promotion interventions. We chose to focus our review on health promotion interventions because of the wide variation of intervention types that exist in this space. Health promotion interventions may include, for example, educational, behavioural, environmental, regulatory and/or structural actions, such as mass media campaigns; legislation and regulation; changes to infrastructure; and community, school, and workplace health and safety programmes. In doing so, we sought to identify key elements of dose that have been monitored during implementation and understand the degree of variation in the application of dose across interventions.

## Methods

A scoping review was used as it allows for rapid mapping of the key concepts underpinning a research area [18]. The methodology for this scoping review was based on the framework outlined by Arksey and O’Malley [19] and ensuing recommendations made by Levac and colleagues [20].

### Identifying relevant studies

We searched four electronic databases, MEDLINE, PsycINFO, Embase and Global Health, to identify articles published in English. See Table 2 for search terms and strategy. Search terms were piloted and refined prior to use, including consultation with experts and checking for capture of studies that the authors expected to be included.

**Table 2:** Overview of search strategy

Search terms	
(intervention OR innovation OR strateg$ OR program OR policy) AND (dos$ OR reach OR exposure OR integrity OR fidelity OR implementation OR uptake OR adoption) AND (measur$ OR defin$ OR concept$ OR performance monitoring OR process evaluation OR program evaluation) AND (health education OR health promotion)	
Search limits	
Published between 2000 and 2015; English Language; Human Contains ‘dose’ or ‘dosage’ in title, abstract or keywords	

### Study selection

After removing duplicates, a total of 4611 articles were identified. Given the large number of results, we decided to further focus the search by identifying a subset of articles that contained the term ‘dose’ or ‘dosage’ within the title, abstract or key words, using a structured field search in Endnote.

The criteria for study inclusion were refined through discussion among the research team as the reviewers became more familiar with the research [20]. A two-stage screening process was used to select studies for inclusion: (1) title and abstract review and (2) full-text review. Articles were included if they were peer-reviewed, original research which used the concept of dose in relation to a health promotion intervention. Studies of medical treatments (e.g. medication, surgery), conference abstracts and dissertations were excluded. Studies of effectiveness were included, as well as studies of diffusion and scale-up, as both types of studies have the opportunity to operationalise dose as part of intervention implementation. Hence, both types of studies can inform ideas about best ways to measure dose.

**Fig. 1:**
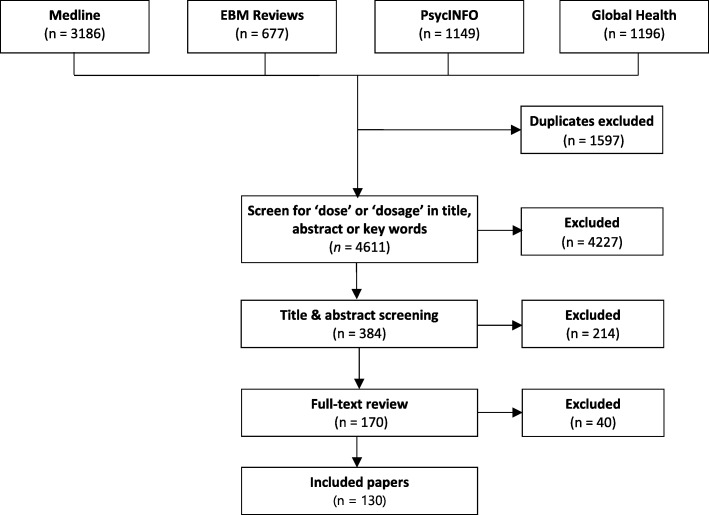
Flow chart of article selection process. Details the flow of information through the different phases of the review and maps out the number of records identified, included and excluded, and the reasons for their exclusion

Title and abstract review was conducted by one reviewer (SR), and a second reviewer (KC) reviewed a random sample of 77 articles (20%) for reliability purposes. Percentage agreement was 95% (Cohen’s kappa *k* = 0.90) and all disagreements were discussed and resolved. Figure 1 outlines the flow of articles through the review process.

### Data extraction and synthesis

We developed a template to extract study characteristics, intervention details, sample, dose terms and definitions and other implementation concepts (see Additional file 1 for data extraction template). The template was pretested on a randomly selected subset of five articles. The characteristics of each full-text article were then extracted by one reviewer, while a second reviewer performed data extraction on a random sample of 20% of articles to check for consistency. There was 91% agreement (*k* = 0.52) between the coders and all disagreements were discussed and resolved.

Extracted data were imported into NVivo qualitative data management software [21] for further coding and synthesis using an inductive approach to identify key patterns in how dose was defined and operationalised across studies, and the original articles were revisited frequently to check interpretations. Data synthesis was performed by one reviewer (SR) and refined through ongoing discussion with other authors.

## Results

### Characteristics of included articles

**Fig. 2:**
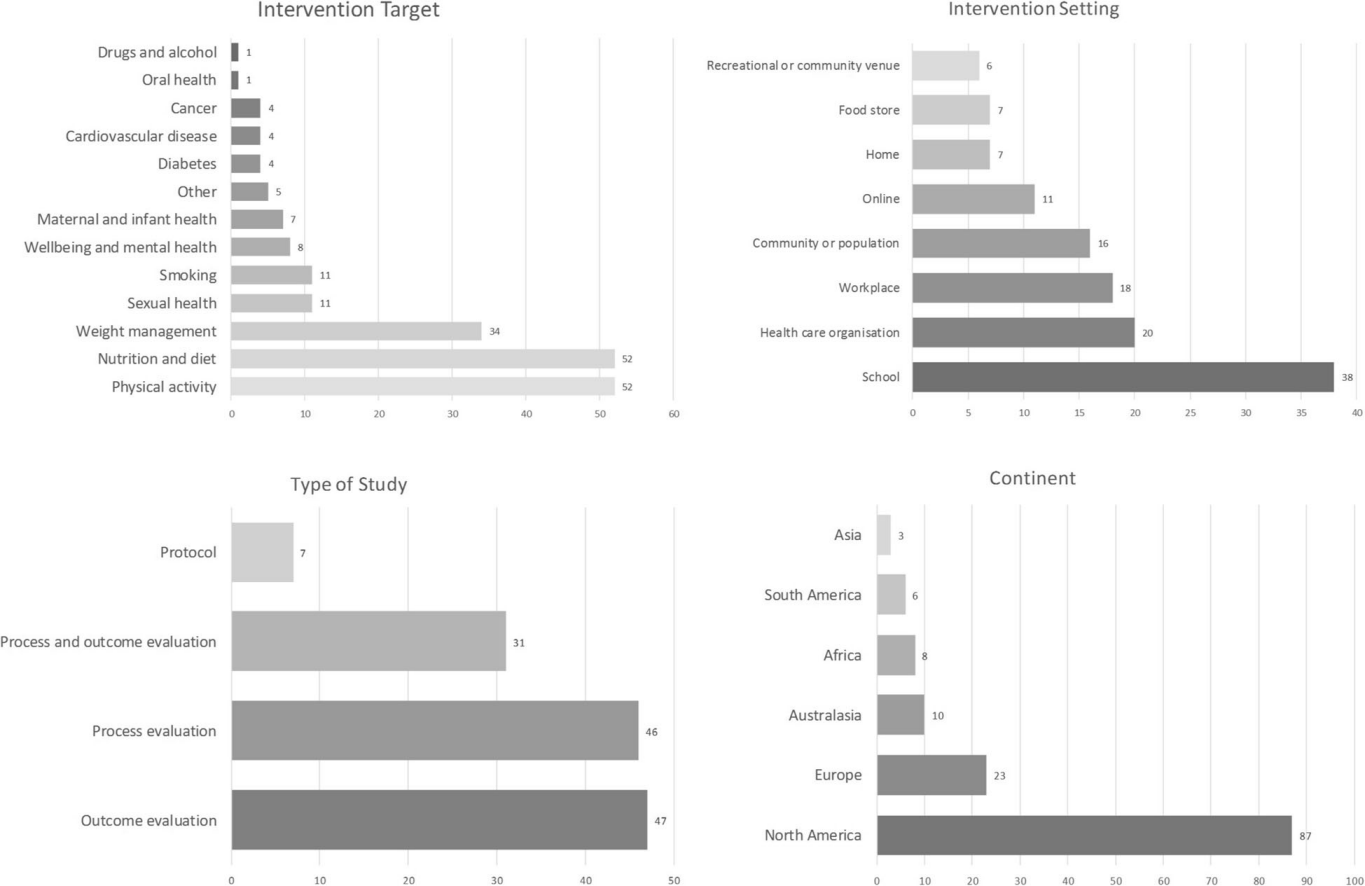
Characteristics of included articles. Bar charts depicting the key characteristics of studies in the sample. From top left to bottom right: (1) intervention target: number of studies targeting each health behaviour or disease; (2) intervention setting: number of studies conducted within each setting; (3) type of study: number of studies that were defined as protocol, process or outcome evaluations; and (4) continent: number of studies conducted in each continent

A total of 130 articles were included in the review. Interventions were conducted across a range of settings, most frequently school, workplace and health care settings. The most common types of interventions were those which aimed to provide information or education or increase awareness about an issue, such as school curriculums, mass media campaigns and information leaflets. Interventions that sought to support behaviour change through strategies such as goal setting, motivational interviewing and counselling were also common in this sample. Less common were interventions providing financial incentives or involving restrictions, regulation or structural and environmental change. See Fig. 2 for a summary of article characteristics and Additional file 2 for details of all included articles.

**Fig. 3:**
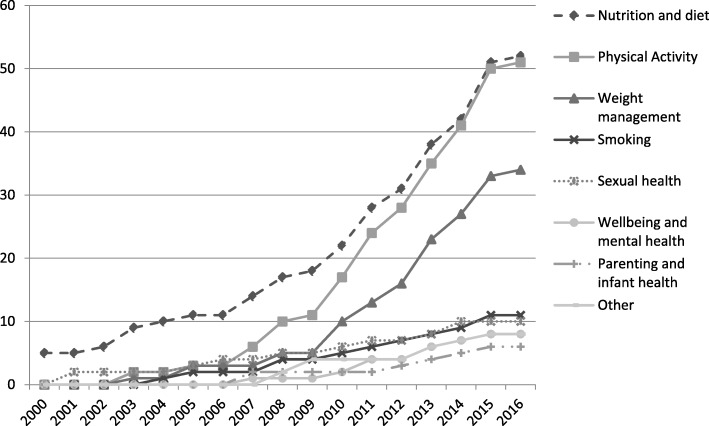
Cumulative frequency of research articles using ‘dose’ over time. Number of studies containing reference to the concept of dose according to health topic. For clarity/ease of interpretation, intervention target categories containing fewer than five studies in total across the sample timeframe (diabetes prevention, drugs and alcohol, oral health, cancer risk, CVD risk) are not shown in the cumulative frequency graph

The number of articles using the concept of dose has increased in recent years, with over two-thirds of the papers in our sample having been published since 2010. Within our sample, physical activity and nutrition were the most frequent intervention targets for studies using the concept of dose. A cumulative frequency chart (see Fig. 3) indicates that the use of ‘dose’ in interventions targeting nutrition and diet, physical activity and weight management has increased considerably in the last decade.

### Variation in terms used to refer to the concept of ‘dose’

Across the sample, there was a range of terms used to refer to dose. Here, we provide a brief overview of these terms and how they were applied. In the subsequent section, we unpack the operationalisation of dose in more depth.

#### Dose and dosage were used somewhat interchangeably

Nearly half of the studies used the term *dose* (*n =* 56), with a handful using the term *dosage* (*n =* 7). It is worthwhile noting that in medicine, the terms ‘dose’ and ‘dosage’ refer to different things: dose refers to the amount of medication (usually measured by weight) given at a single time, while dosage refers to amount of medication per unit of time (i.e. the rate at which dose is administered) and implies a medication regimen rather than a single administration. Within our sample, these terms were used somewhat interchangeably and did not align with the definitions used in medicine. In particular, the term ‘dose’ was used in a variety of ways, sometimes to refer to the amount of intervention at a single time point, but often also to refer to the delivery or receipt of the intervention over time. For example, Ayala and colleagues [22] measured ‘intervention dose’ as ‘the number of classes each community participant attended between baseline and 6 months and between 6 and 12 months’ (p. 2264). The term ‘dosage’ was generally used to refer to the proportion, duration or frequency of intervention components over the intervention period. For example, Fagan and colleagues [23] defined programme dosage as ‘the extent to which programs achieved the required number, length, and frequency of sessions’ (p. 244).

#### Dose delivered and dose received were well differentiated

The terms *dose delivered* (*n =* 33) and *dose received* (*n =* 28) were commonly used, frequently appearing together as differentiated concepts within the same papers (*n =* 27). For example, Curran and colleagues [24] defined dose delivered as ‘the amount of intended units of each intervention component provided to target audience’ and dose received as ‘the extent of engagement of participants’ (p. 721).

#### Dose-response was captured in about half of the studies

Sixty-four studies (49%) went beyond simply defining and measuring dose and linked their measure of dose to the outcomes of the study. For example, in their evaluation of phone-based weight loss programmes, Sherwood and colleagues [25] defined dose as the number of telephone counselling sessions provided and reported that more counselling calls were associated with greater weight loss and a higher frequency of engaging in weight-related self-monitoring behaviours. It was most common to use the term ‘dose-response’ although three studies used the term ‘dose-effect’ to mean the same thing.

#### Some studies developed new terms to capture particular aspects of dose

Occasionally, new terms were introduced to identify particular aspects of dose. For example, two studies [26, 31] used the term ‘population dose’ to refer to the effect size of an intervention, calculated as intervention reach multiplied by effect size for each person. This measure of dose attempts to capture the impact of interventions delivered at the population level, where traditional measures of dose may fall short.

Other terms used to refer to dose included ‘minimum dose sample’ to refer to a sample of participants who participated to a minimum extent [27] and ‘dose intensity’ which was defined as the relative difference in intensity of dose across similar versions of the same intervention [28] In this case, a ‘higher dose intensity’ in a church-based lifestyle intervention involved adding, for example, follow-up phone calls, increasing the number of sessions and providing on-site equipment.

Wasik and colleagues [12] argued that dosage must be considered on two levels: (1) the intervention staff learning new skills and (2) the target group (intended beneficiaries). They suggest that one component of what they call *implementation dosage* ‘refers to implementation activities necessary for intervention to be carried out with fidelity’. They further suggest that another necessary consideration is what they call *intervention dosage* which is the amount of intervention provided that is necessary to change the target group’s behaviour. These factors, they argue, are critical for cost, staffing, replication and scale-up considerations. However, the new term is an umbrella for several variables: a conventional view of dose (amount of an intervention), its delivery over time, its potency (capacity to bring about an effect) and interaction with context. The term implies a critical threshold level of dosage. Critical levels of the other factors need to be examined as well.

### Variation in how dose was operationalised

While most studies provided a definition of dose and/or detail on how it was operationalised, there was some variation in how this was presented. Table 3 presents an overview of how dose has been operationalised across studies. The main way in which dose was operationalised was in terms of the amount of intervention delivered and/or received, with these aspects being relatively well delineated. However, in some instances, there was an apparent conflation of dose with other concepts such as fidelity to planned intervention and participant satisfaction. These aspects are unpacked in more detail below.

**Table 3:** Overview of the key ways in which the terms dose, dose delivered and dose received were conceptualised across the included intervention studies

Operationalisation	Dose or dosage	Dose delivered	Dose received
Planned intervention			
Amount of intervention planned (e.g. number of intervention components planned; intended length of intervention components)	✓		
Intervention delivery			
Number of intervention components delivered	✓	✓	
Length/duration of intervention components	✓	✓	
Frequency of delivery of intervention components	✓	✓	
Completeness of intervention delivery (amount of intervention components delivered as a proportion of those planned)	✓	✓	
Intensity of intervention	✓		
Availability of intervention		✓	✓
Content of intervention components (and degree to which these were delivered as planned)		✓	
Intervention receipt			
Number of intervention components received	✓		✓
Exposure	✓		✓
Reach	✓		
Attendance	✓		✓
Completion of intervention activities			✓
Use of intervention materials			✓
Extent of engagement with intervention	✓		✓
Other concepts			
Number of people trained to deliver intervention	✓		
Commitment to intervention		✓	✓
Satisfaction with intervention	✓		✓
Perceptions of intervention feasibility			✓

Note: While some of the concepts in this table would perhaps not usually be considered to be part of dose, this synthesis is based on a consideration of how dose was defined and operationalised in practice across the included articles. We explore the implications for these conceptualisations of dose in the ‘Discussion’ section

#### Dose as intervention delivered

Consistent with the frameworks outlined in Table 1, a large proportion of studies operationalised dose in terms of the *amount of intervention delivered*, considered as a function of the efforts of intervention providers. Most frequently, this was captured using measures such as the number of intervention components delivered (e.g. number of lessons, posters and stickers delivered; [29]), frequency or duration of intervention components (e.g. lesson length; [30]) and time spent on intervention activities [31]. For example, Baquero and colleagues [32] measured dose delivered as the number and length of home visits completed, Hall and colleagues [33] as the duration of education classes delivered, and Rosecrans and colleagues [8] as the number of intervention materials (food samples, flyers and recipes distributed).

A handful of studies operationalised dose as the *intensity* of intervention delivery by creating discrete categories. For example, Koniak-Griffin and colleagues [34] considered the effect of ‘treatment dosage (intensity)’ such that ‘participants were classified into two categories (low/medium and high intensity levels)’ (p. 80) based on class attendance and teaching and coaching contacts received, while Rubinstein and colleagues [35] examined ‘potential changes in outcomes with increasing intensity (dose) of the intervention’ (p. 56).

Some studies also considered *completeness* of intervention delivery, where the number of intervention components delivered was considered as a proportion of those planned (e.g. [23, 31, 36]). One school-based study called this ‘fidelity to classroom dose’ and while time spent on each activity was also measured, it was referred to as ‘duration’, not dose [37]. Given that fidelity of an intervention might manifest in a number of ways (e.g. teaching quality, information accuracy), this non-conventional conflating of fidelity and dose together as a term could mislead readers who do not take care to read the details of author’s methods. But it was not uncommon; a number of studies used *composite* measures of dose that incorporated a range of elements including aspects of quality of communication. For example, in their evaluation of a school-based tobacco intervention, Goenka and colleagues [38] refer to ‘dose given (completeness)’, which they define as ‘the *quantity* and *rigour* of implementation of the intended intervention units that are actually delivered to the participants’ (p. 925, italics added). This is calculated as a composite score across a range of variables, including percentage of classroom sessions and intra-session elements delivered, proportion of posters displayed, proportion of postcards delivered, participation in an inter-school event, proportion of teachers and student peer leaders trained to deliver the intervention and proportion of sessions where teachers and peer leaders communicated well [38]. As such, their conceptualisation of ‘dose given’ contains a number of different elements of dose related not only to *quantity* of dose delivered, but also *quality of delivery* and *implementation dose* (i.e. training to deliver the intervention). These authors then investigated which aspects were most strongly associated with programme effects. Quality of delivery and implementation dose would typically be considered to be distinct from dose delivered, so referring to these together within the composite term ‘dose given’ could potentially mislead the reader.

#### Dose as intervention received

Another common way in which dose was operationalised was in terms of the amount of intervention received by participants, which was generally captured in one of two ways, implying either an active or passive role for participants. Measures of dose that implied a more passive role for participants included the number of *intervention components received* by participants (such as telephone calls, leaflets, home visits; e.g. [39–41]). A number of studies also used the concepts of *exposure* and *reach*. For example, Lee-Kwan and colleagues [42] conducted a modified Intervention Exposure Assessment survey to assess whether people had seen the intervention materials (within a retail setting). In the context of media campaigns, both Farrelly and colleagues [43] and Huhman and colleagues [44] referred to dose in terms of *reach* or *exposure* to the campaign, to examine the dose-response. Similarly, Birnbaum and colleagues [45] indicated that participants could be identified as ‘belonging to one of four exposure groups (or “doses”): (1) control group: lowest exposure, (2) school environment interventions only, (3) classroom curriculum plus school environment interventions, and (4) peer leaders plus classroom curriculum plus school environment interventions: highest exposure’ (p. 428). Here, dose was defined according to the logic of the intervention designers, with some categories being reasoned to be lower or higher than another in terms of what appears to be the intervention’s penetration into different ecological levels (i.e., organisational level versus group level).

Concerning measures of dose that implied a more active role for participants, Steckler and Linnan [11] define dose received as the extent to which participants engage or interact with, are receptive to or use an intervention, such that dose received is a function of the actions of intervention participants. Examples from studies in our sample included those measuring attendance and participation rates [46, 47], time spent engaged in intervention activities [48], use of intervention components [49] and completion of intervention activities [8, 50–52]. A number of studies also considered dose received in terms of the extent to which participants actively engaged with intervention components [38, 47, 49]. One study also measured the amount of intervention received in terms of the number of activities *remembered* by participants at the end of the intervention [31]. The last example could be considered problematic if used on its own as, cognitively speaking, remembering an activity is a step beyond being exposed to it and programme effects may or may not be captured through conscious recall processes. However, the school-based health education programme being assessed in that study also used observer rating of lessons delivered, and teacher reports of components delivered to assess differences in participant-perceived dose and dose as measured by observers and ‘deliverers’ [31].

As with dose as intervention delivered, some studies conflated dose received with other implementation concepts. For example, Berendsen and colleagues [53] studied a lifestyle intervention in primary care settings and ‘dose received was defined as participant satisfaction and perception of the program that was delivered to them’ (p. 4). While the latter idea (perception that the programme was delivered) would commonly be used as a way of tapping into dose received, the former (satisfaction) might be thought of as something separate. This illustrated a tendency in the literature to characterise dose in terms of the extent to which the intervention met a particular criteria. Another study [54] clearly makes a conceptual distinction between dose received and satisfaction throughout the paper, but measuring and listing them together, i.e. as ‘dose received (exposure and satisfaction)’ (p. 76) could mislead readers into thinking they were treated as one and the same.

One investigator team reported on participants’ ‘evaluation of dose received’ [55] and defined this as the participants’ views about intervention feasibility. This is not conflating the definition of feasibility with dose. Evaluation involves two processes: (1) observation/measurement and (2) judgement [56]. By declaring that they are ‘evaluating’, both processes are assumed and indeed in this study, both processes were performed. However, the use of the words ‘evaluating’ dose as opposed to ‘measuring’ dose could mislead readers not familiar with these distinctions from the field of evaluation to think there is definitional slip about dose.

#### A small proportion of studies referred to ‘dose’ but did not operationalise it in their methods

While most studies provided some detail on how dose was measured, 15 (11.5%) studies referred to dose without any further detail. Of these, most used the term dose when interpreting their results but did not refer to the measurement or operationalisation of dose within their methods or findings. For example, Kelishadi and colleagues [57] state that ‘the overall lower increase of junk food consumption … showed that the dose or intensity of our community-based and school-based interventions, although not sufficient, was necessary to act against other forces in the community’ [57] but did not refer to the measurement of dose elsewhere within the main body of the paper. The effect was that it was not clear what was actually considered the dose of the intervention (i.e. what components and how much of these was considered to have brought about the observed effect).

## Discussion

We have presented the results of a scoping review exploring how dose is defined and operationalised in the implementation and evaluation of health promotion interventions. The findings suggest that there are some commonalities in how dose was defined and operationalised across studies, with studies most commonly focusing on measurements of (1) the *amount of intervention delivered*, where dose is conceptualised as a function of intervention providers and the focus is on the supply side of the intervention and (2) the *amount of intervention received*, where the focus is on how much of the intervention the recipients’ actually get, sometimes conceptualised as a function of the actions of recipients (e.g. whether they attend sessions or collect intervention materials). Most of the studies within this review measured dose in some form and in that sense, we note that they complied with the Template for Intervention Description and Replication (TIDieR) guidelines for intervention reporting (Item 8: ‘when and how much’) [58].

However, in many instances, it would not have been easy for study authors to fit health promotion interventions into the conceptual framing of dose delivered and dose received. While some aspects of health promotion interventions allow for such a separation, for example, dose delivered as the number of intervention materials (e.g. leaflets, posters) displayed within an intervention site or dose received as the number of intervention materials taken away by participants, the distinction becomes somewhat arbitrary for components that concern participant interaction and engagement (such as team competitions) [52]. Similarly, the separation of components like phone calls and home visits (i.e. events for which delivery and receipt are inseparable) into categories of ‘delivered and received’ could seem somewhat artificial. However, despite these difficulties, the distinction between dose delivered and dose received is important. In a notable example, Wilson and colleagues demonstrated that their intervention achieved moderate to high levels of dose delivered and only moderate levels of dose received and that the former was better than the latter in terms of achieving health outcomes. This was sufficient for them to recommend to others replicating their intervention to spend more time making sure components of interventions are delivered and to worry less about people completing all the activities as directed [52].

All studies were limited by what was measurable and hence investigators may have had to devise a proxy for what might have been their preferred way to measure dose. Within environmental interventions dose measurement is often based on recall or self-report of what people saw (e.g. [42]) although this is not, strictly speaking, the same as being exposed. Identifying what should be measured in order to capture dose is also likely to be problematic when considering population-level policy interventions, such as increasing the amount of urban green space in order to improve health [59]. For example, should ‘dose’ be counted as the number of new trees planted, amount of coverage of green space relative to size of neighbourhood or something else? Traditional conceptualisations of dose as intervention components delivered and received are not easily applied to such interventions. Such considerations have important implications for how interventions are planned, resourced and delivered, highlighting the need for critical thought about how we capture the ‘dose’ of population level interventions that go beyond ‘traditional’ health promotion interventions. While our review highlighted some novel attempts to capture dose, we may need to look more to disciplines like geography and political science in order to further the notion of dose as applied to population-level policy interventions.

Unfortunately, our capacity to review innovative approaches to how policy and environmental interventions in health promotion are addressing ‘dose’ may have been limited by our choice of ‘dose’ and ‘dosage’ as our primary search terms. For example, if a study assessed the impact of policy exposure without using the word dose, our search would not have captured this. This was the chief limitation of our study design and is a clear priority area for further investigation. Progress in the scanning and benchmarking of international obesity policy could provide an example in this direction [60].

A few investigators expanded on the traditional concept of dose, seeking to combine it with other elements to tell a story that is more than just amount received or delivered. As discussed previously, Wasik and colleagues [12] developed the term *implementation dosage* to refer to activities needed to achieve fidelity and intervention dosage (defined as the amount of intervention needed to change the target group’s behaviour). In a similar vein, Cheadle and colleagues [13, 26] offer the concept of population dose, defined as ‘the estimated community-level change in the desired outcome expected to result from a given community-change strategy, operationalized as the product of penetration (reach divided by target population) and effect size’ [13]. This approach has been applied to measurement of the impact of community, organisation and school level policy and environmental changes to improve physical activity and nutrition [26, 61]. Cheadle reports that he and his team first ‘tested’ the lay understanding of dose with health department practitioners, community groups, funders and the federal government and decided to build on it because it conveys the notion of ‘active ingredient’ as something that makes something happen [15]. The ideas of both Wasik et al. and Cheadle et al. draw attention to the dynamics of the change process and the role of dose in the change process. The idea of ‘intervention dose’ (although Wasik et al. use ‘dosage’) has caught on, with researchers now applying it to fields like (medical) quality improvement where they define intervention dose as quantity, exposure, intensity, scope, reach, engagement, duration and quality.

In a sense, therefore, ‘dose’ has become the gateway for the appreciation of factors that others would consider to be part of process evaluation, such as fidelity or rigour [11]. Whether this is a problem depends on whether these conceptualisations of dose lead to an over-simplification of ‘what’ has to be transported for place-to-place achieve effects. That is, if the measurement of dose overemphasises the intervention at the expense of understanding contextual dynamics. Cotterill and colleagues have recently suggested changes to the TIDieR guidelines in acknowledgement that all aspects of an intervention can change over time and researchers need to be alert to differences arising through adaptation [62]. A population health and policy version of the checklist has since been launched which, in addition to tracking planned and unplanned variation, calls for the date and duration of intervention to be reported [63]. Researchers have also been urged to fully appreciate the complex, non-monotonic ways in which dose response is brought about [64]. This implies a need to not ‘over assume’ the criticality of dose, *vis-à-vis* other options, such as the critical threshold aspects within the context (e.g. demographics, interactivity, existing resources).

Another implication is that researchers should measure dose in multiple ways and fully interrogate the meaning of the results. An exemplar in this regard is the work by Goenka and colleagues [38] in evaluating a school-based tobacco prevention programme in schools in India. They found that the proportion of teachers trained to deliver the intervention was a better predictor of the results than time spent on the intervention. The findings point to the possibility of a multi-strand pathway of change in school settings where, in this instance, the proportion of teachers trained acts as an alternative or additional casual pathway to health outcomes [16]. This idea invites the use of complicated and/or complex logic models in intervention evaluation where the ‘dose’ of multiple factors, and the way each interacts with the others, become critical considerations [65]. Interactions among causal factors might explain non-linear, dose-response relationships where the same ‘amount’ delivered at different times in an intervention’s history or in different contexts has different proportional effects on outcome, because of the cumulative effects of reinforcing feedback loops and/or the existence of threshold effects or ‘tipping points’ [65].

## Conclusion

It seems vital that researchers in health promotion continue to measure dose in multiple ways, and further explore how to define and measure the dose of population-level interventions and policies. We suggest researchers also reverse their tendency to use ‘dose’ and ‘dosage’ interchangeably. Dosage’s focus on rate of delivery over time requires researchers to monitor response to ‘dose’ and adjust accordingly. It embraces the complexity of intervention design and evaluation that is now being more widely acknowledged [66, 16].

## Additional files

Additional file 1: Data extraction template with example data extraction. (DOCX 15 kb)

Additional file 2: Article characteristics. (DOCX 183 kb)

## Abbreviations

TIDieR: Template for Intervention Description and Replication
