# Supplementary material for: Variation in the operationalisation of dose in implementation of health promotion interventions: insights and recommendations from a scoping review
Source: Implement Sci. 2019 Jun 6;14:56. doi: 10.1186/s13012-019-0899-x (PMC6555031; doi:10.1186/s13012-019-0899-x)
Supplement: Supplementary file 1 — Data extraction template with example data extraction. (DOCX 15 kb) [file 13012_2019_899_MOESM1_ESM.docx]

| **Supplementary File 1. Data extraction template with example data extraction** | | | |
| --- | --- | --- | --- |
| **Field** | **Notes** | **Example 1** | **Example 2** |
| **Author** |  | Curran, et al. | Freedman, et al. |
| **Year** |  | 2005 | 2013 |
| **Title** |  | Process evaluation of a store-based environmental obesity intervention on two American Indian Reservations | A farmers' market at a federally qualified health centre improves fruit and vegetable intake among low-income diabetics |
| **Include/Exclude** | Include OR Exclude | Include | Include |
| **Study Type** | Empirical OR Review OR Theoretical OR N/A | Empirical | Empirical |
| **Country** | Country in which the study was conducted  (If multiple countries for data collection then list all here) | US | US |
| **Paper type** | Process evaluation OR Outcomes OR Other (specify) | Process evaluation | Outcome evaluation |
| **Intervention Design** | What is the design of the intervention  (e.g. RCT, pre-post, longitudinal). | Non-randomised trial: Intervention vs. comparison, with pre-post assessment and longitudinal follow up | Community-based participatory research. One-group repeated measures (baseline, mid-intervention, post-intervention) |
| **Intervention target** | What is the intervention targeted to address  (e.g. obesity, alcohol, wellbeing, exercise, smoking cessation, reduction in alcohol) | Reducing obesity (by increasing access to healthy food) | Increasing fruit and veg (FV) consumption |
| **Intervention participants** | Who are the target recipients of the intervention? (e.g. school employees, hospital employees) | Apache reservation residents | Health centre patients with diabetes |
| **Intervention setting** | What is the target setting for the intervention? (e.g. community, workplace). | Community (food stores in Apache reservations) | Community (farmers market at healthcare centre) |
| **Intervention components** | List the key components of the intervention. If multiple intervention conditions then specify this and list separately | Whole community intervention. List of promoted foods to be stocked; In-store (shelf labels and posters) and mass-media (newspaper cartoons, radio broadcasts) strategies to communicate behavioural messages; Cooking demos and taste tests | 1. Weekly farmers market at health centre for 22 weeks (accepted supplemental nutrition assistance program (SNAP) vouchers), 2. Financial incentives (uo to $50 in vouchers) to purchase FV |
| **Dose terms** | List the dose terms used within the paper, (e.g. dose delivered, dose received, dose-response) | Dose delivered; Dose received | Dose-response |
| **Dose definition** | Provide the definition for each of the dose terms listed in the paper. | **Dose delivered**: Amount of intended units of each intervention component provided to target audience. **Dose received:** Extent of engagement of participants | None given |
| **Dose operationalization** | Describe how each of the dose terms have been operationalized in relation to the intervention components, (e.g. % of activities provided relative to those planned) | **Dose delivered:** Number of demos delivered; Average length of demos; Number of times cartoons were published/radio ads were broadcast; Average number of food samples, flyers and recipe cards delivered at food demo. **Dose received:** Average number of food samples, flyers and recipe cards delivered at food demo | Number of visits to farmers market |
| **Methods of dose data collection** | What methods were used to collect data on dose? (e.g., self-report by participants, observation by intervention staff, observations by process evaluator). What tools/instruments were used to record dose? (e.g. computer program, record forms etc) | **Observations completed by full time independent process evaluator** [Dose delivered: Store visit evaluation form, mass media log, observation of in-store cooking demonstrations; Dose received: observation of in-store cooking demonstrations] | Receipt of each sales transaction (matched to participant ID) (n = 45) |
| **Links dose & outcome?** | Does the paper link dose with outcomes? | No | Yes. Dose-response used as variable in analysis |
| **Results of link between dose and outcomes** | If the paper dose link dose with outcomes then provide the key findings here | N/A | The odds of increasing FV consumption were higher for diabetics who visited the FM more often |
| **Comments** | Provide any relevant comments pertaining to dose. Note if the definition or reporting of dose overlaps or is conflated with related terms such as reach, fidelity etc. | Dose is not clearly operationalised and is often talked about in conjunction with fidelity such that the two are not clearly distinguishable. |  |
