# Supplementary material for: Variation in the operationalisation of dose in implementation of health promotion interventions: insights and recommendations from a scoping review
Source: Implement Sci. 2019 Jun 6;14:56. doi: 10.1186/s13012-019-0899-x (PMC6555031; doi:10.1186/s13012-019-0899-x)
Supplement: Supplementary file 2 — Article characteristics. (DOCX 183 kb) [file 13012_2019_899_MOESM2_ESM.docx]

| **Supplementary File 2.** Article characteristics | | | | | | | |
| --- | --- | --- | --- | --- | --- | --- | --- |
|  | **Author** | **Year** | **Country** | **Intervention target** | **Intervention setting** | **Intervention type** | **Dose terms used** |
| [1] | Aarestrup, et al. | 2015 | Denmark | Diet and nutrition | School | Environmental | Dose delivered; Dose received |
| [2] | Alia, et al. | 2015 | US | Weight management | Community | Behavioural | Dose delivered; Dose received |
| [3] | Androutsos, et al. | 2014 | Belgium, Bulgaria, Germany, Greece, Poland, Spain | Diet and nutrition; Physical activity | School | Environmental; Capacity building | Dose delivered; Dose received |
| [4] | Ayala | 2011 | US | Physical activity | Community | Behavioural | Dose or dosage |
| [5] | Baquero, et al. | 2014 | US | Diet and nutrition | Food store | Information, education or awareness; Environmental; Capacity building | Dose delivered; Dose received |
| [6] | Baquero, et al. | 2009 | US | Diet and nutrition | Home | Environmental | Dose delivered; Dose received |
| [7] | Baranowski, et al. | 2003 | US | Diet and nutrition; Physical activity | Recreation or community venue | Behavioural | Dose or dosage |
| [8] | Bellows, et al. | 2013 | US | Physical activity | School | Information, education or awareness | Dose or dosage |
| [9] | Berendsen, et al. | 2015 | Netherlands | Weight management; Diet and nutrition; Physical activity | Healthcare organisation | Behavioural | Dose delivered; Dose received |
| [10] | Beresford, et al. | 2000 | US | Diet and nutrition | Workplace | Environmental | Dose delivered |
| [11] | Berkowitz, et al. | 2008 | US | Physical activity | Community | Information, education or awareness | Dose or dosage |
| [12] | Binkley, et al. | 2014 | US | Oral health | Healthcare organisation | Environmental; Capacity building; Behavioural | Dose or dosage |
| [13] | Birnbaum, et al. | 2002 | US | Diet and nutrition | School | Information, education or awareness; Environmental; Behavioural | Dose or dosage |
| [14] | Bishop, et al. | 2013 | US | Drugs and alcohol | School | Information, education or awareness | Dose or dosage |
| [15] | Bjelland, et al. | 2011 | Norway | Weight management; Diet and nutrition; Physical activity | School | Information, education or awareness; Environmental | Dose received |
| [16] | Bolier, et al. | 2013 | Netherlands | Wellbeing and mental health | Online | Information, education or awareness; Behavioural | Dose-response |
| [17] | Bowen, et al. | 2008 | US | Sexual health | Online | Information, education or awareness | Dose-response |
| [18] | Branscum, et al. | 2013 | US | Weight management | School | Behavioural | Dose delivered; Dose received |
| [19] | Broekhuizen, et al. | 2012 | Netherlands | Weight management; Parenting and infant health | Healthcare organisation | Information, education or awareness; Behavioural | Dose or dosage |
| [20] | Buckley & Sheehan | 2009 | Australia | Other: Adolescent risk taking and injury | School | Information, education or awareness | Dose or dosage |
| [21] | Buller, et al. | 2005 | US, Canada | Cancer | Workplace | Information, education or awareness; Environmental | Dose-response |
| [22] | Buller, et al. | 2008 | Australia & US | Smoking | School | Information, education or awareness | Dose-response |
| [23] | Caldera, et al. | 2007 | US | Maternal and infant health | Healthcare organisation | Behavioural | Dose or dosage |
| [24] | Campbell, et al. | 2007 | US | Diet and nutrition | Faith organisation | Information, education or awareness; Environmental; Behavioural | Dose or dosage |
| [25] | Campbell, et al. | 2004 | US | Diet and nutrition | Healthcare organisation | Information, education or awareness | Dose or dosage |
| [26] | Cargo, et al. | 2006 | Canada | Diabetes | School | Information, education or awareness | Dose or dosage |
| [27] | Cheadle, et al. | 2012 | US | Diet and nutrition; Physical activity | Community | Environmental | Other: Population dose |
| [28] | Chen, et al. | 2012 | US | Smoking | Community | Information, education or awareness; Environmental | Dose-response |
| [29] | Chittleborough, et al. | 2013 | UK | Other: Hand washing | School | Information, education or awareness | Dose or dosage |
| [30] | Coffeng, et al. | 2013 | Netherlands | Physical activity; Wellbeing and mental health | Workplace | Environmental; Capacity building; Behavioural | Dose delivered; Dose received |
| [31] | Cook, et al. | 2007 | US | Diet and nutrition; Physical activity; Wellbeing and mental health | Workplace; Online | Information, education or awareness | Other: Dose effects |
| [32] | Cook, et al. | 2015 | US | Diet and nutrition; Physical activity; Wellbeing and mental health | Workplace; Online | Information, education or awareness | Other: Dose effects |
| [33] | Coulon, et al. | 2012 | US | Weight management; Physical activity | Community | Information, education or awareness; Behavioural | Dose or dosage |
| [34] | Cowan & Devine | 2012 | US | Weight management; Diet and nutrition | Healthcare organisation | Information, education or awareness; Environmental | Dose delivered |
| [35] | Crutzen, et al. | 2013 | Netherlands | Sexual health | Online | Information, education or awareness | Dose or dosage |
| [36] | Curran, et al. | 2005 | US | Weight management; Diet and nutrition | Food store | Information, education or awareness; Environmental | Dose delivered; Dose received |
| [37] | Day, et al. | 2008 | Canada | Weight management; Diet and nutrition | School | Economic or financial; Capacity building; Behavioural | Dose or dosage |
| [38] | Devine, et al. | 2012 | US | Diet and nutrition; Physical activity | Workplace | Environmental; Behavioural | Dose delivered; Dose received |
| [39] | DiClemente, et al. | 2014 | US | Sexual health | Healthcare organisation | Behavioural | Dose or dosage |
| [40] | Fagan, et al. | 2008 | US | Other: Adolescent problem behavior | Community | Capacity building | Dose or dosage |
| [41] | Farrelly, et al. | 2005 | US | Smoking | School | Information, education or awareness | Dose or dosage |
| [42] | Fort, et al. | 2015 | Mexico & Costa Rica | Diet and nutrition; Physical activity; Cardiovascular disease | Healthcare organisation | Information, education or awareness | Dose-response |
| [43] | Fotu, et al. | 2011 | Tonga | Weight management; Diet and nutrition; Physical activity | Community | Information, education or awareness; Capacity building | Dose or dosage |
| [44] | Freedman, et al. | 2013 | US | Diet and nutrition | Healthcare organisation; Community | Economic or financial; Environmental | Dose-response |
| [45] | Gittelsohn, et al. | 2013 | US | Weight management; Diet and nutrition; Cardiovascular disease | Recreation or community venue; Food store | Information, education or awareness; Environmental; Behavioural | Dose or dosage |
| [45, 46] | Gittelsohn, et al. | 2010 | US | Diet and nutrition | Food store | Environmental | Dose or dosage |
| [47] | Goenka, et al. | 2010 | India | Smoking | School | Information, education or awareness | Dose delivered; Dose received |
| [48] | Goode, et al. | 2011 | Australia | Diet and nutrition; Physical activity | Home | Information, education or awareness; Behavioural | Dose or dosage |
| [49] | Griffin, et al. | 2010 | US | Physical activity | Community | Behavioural | Dose or dosage |
| [50] | Hall, et al. | 2012 | US | Physical activity; Diabetes | School | Information, education or awareness | Dose delivered; Dose received |
| [51] | Havas, et al. | 2003 | US | Diet and nutrition | Healthcare organisation | Information, education or awareness; Behavioural | Dose-response |
| [52] | Heelan, et al. | 2015 | US | Weight management | School | Environmental | Other: Population dose |
| [53] | Hillemeier, et al. | 2008 | US | Maternal and infant health | Healthcare organisation | Information, education or awareness; Behavioural | Dose-response |
| [54] | Hoekstra, et al. | 2014 | Netherlands | Physical activity | Healthcare organisation | Behavioural | Dose delivered; Dose received |
| [55] | Huhman, et al. | 2007 | US | Physical activity | Community | Information, education or awareness | Dose or dosage; Dose-response |
| [56] | Huhman, et al. | 2010 | US | Physical activity | Community | Information, education or awareness | Dose-response |
| [57] | Hunt, et al. | 2000 | US | Diet and nutrition | Healthcare organisation | Information, education or awareness; Environmental | Dose-response |
| [58] | Hwang, et al. | 2013 | US | Cancer | Online | Information, education or awareness | Other: Minimum dose sample |
| [59] | Johnson, et al. | 2010 | US | Weight management | Workplace | Environmental; Behavioural | Dose or dosage |
| [60] | Kaphingst, et al. | 2007 | US | Diet and nutrition; Physical activity; Cancer | Healthcare organisation | Behavioural | Dose or dosage |
| [61] | Kaufman, et al. | 2013 | Tanzania | Sexual health | Community | Information, education or awareness | Dose-response |
| [62] | Keating, et al. | 2015 | Australia | Physical activity | Not specified | Behavioural | Dose or dosage |
| [63] | Kelishadi, et al. | 2011 | Iran | Diet and nutrition; Smoking; Physical activity | Community | Information, education or awareness; Environmental | Dose or dosage |
| [64] | Kim, et al. | 2015 | Ethiopia | Maternal and infant health | Community | Information, education or awareness; Capacity building; Behavioural | Dose delivered; Dose received |
| [65] | Knowlden & Sharma | 2014 | US | Weight management | Online | Information, education or awareness | Dose delivered; Dose received |
| [66] | Koniak-Griffin, et al. | 2015 | US | Weight management; Diet and nutrition; Physical activity | Community | Behavioural | Dose or dosage |
| [67] | Kozica, et al. | 2015 | Australia | Weight management | Community | Information, education or awareness | Dose delivered |
| [68] | Kulwa, et al. | 2014 | Tanzania | Maternal and infant health | Healthcare organisation | Information, education or awareness; Behavioural | Dose delivered; Dose received |
| [69] | Lane, et al. | 2015 | Ireland | Physical activity | Community | Information, education or awareness; Behavioural | Dose or dosage |
| [70] | Lee, et al. | 2013 | US | Smoking | School | Environmental | Dose-response |
| [71] | Lee, et al. | 2015 | US | Diet and nutrition | Food store | Economic or financial; Environmental | Dose or dosage |
| [72] | Lee-Kwan, et al. | 2013 | US | Diet and nutrition | Food store | Environmental | Dose delivered; Dose received |
| [73] | Lemon, et al. | 2010 | US | Weight management | Workplace | Information, education or awareness; Environmental; Behavioural | Dose-response |
| [74] | Magnani, et al. | 2005 | South Africa | Sexual health | School | Information, education or awareness | Dose-response |
| [75] | Marcus, et al. | 2015 | US | Physical activity | Online | Information, education or awareness; Behavioural | Dose or dosage |
| [76] | Mathews, et al. | 2010 | Australia | Weight management | School | Environmental; Capacity building | Dose or dosage |
| [77] | McAlister, et al. | 2004 | US | Smoking | Community | Information, education or awareness; Environmental | Dose or dosage |
| [78] | McCreary, et al. | 2010 | Malawi | Sexual health | Healthcare organisation; Recreation or community venue | Information, education or awareness | Dose delivered; Dose received |
| [79] | Mitchell, et al. | 2015 | US | Weight management; Diet and nutrition; Physical activity | Workplace | Information, education or awareness | Dose-response |
| [80] | Murrock & Gary | 2010 | US | Weight management; Physical activity | Faith organisation | Behavioural | Dose or dosage |
| [81] | Myers, et al. | 2014 | US | Weight management | Not specified | Behavioural | Dose or dosage |
| [82] | Nassau, et al. | 2013 | Netherlands | Weight management | School | Information, education or awareness; Environmental | Dose or dosage |
| [83] | Nathan, et al. | 2010 | Australia | Wellbeing and mental health | Community; Recreation or community venue | Capacity building; Behavioural | Dose-response |
| [84] | Nicklas & O'Neil | 2000 | US | Diet and nutrition | School | Information, education or awareness; Environmental | Dose or dosage |
| [85] | Nicklas, et al. | 2013 | US | Weight management; Physical activity | School | Information, education or awareness | Dose or dosage |
| [86] | Nicklas, et al. | 2011 | US | Diet and nutrition | School | Information, education or awareness | Dose or dosage |
| [87] | O'Hara, et al. | 2011 | Australia | Weight management; Diet and nutrition; Physical activity | Community | Information, education or awareness | Dose-response |
| [88] | Ostwald, et al. | 2014 | US | Other: Stroke recovery | Home | Information, education or awareness; Behavioural | Dose or dosage |
| [89] | Oude Hengel, et al. | 2011 | Netherlands | Wellbeing and mental health | Workplace | Capacity building; Behavioural | Dose delivered; Dose received |
| [90] | Parker, et al. | 2010 | US | Weight management | Workplace | Information, education or awareness; Environmental; Behavioural | Dose or dosage |
| [91] | Paul & Olson | 2013 | US | Weight management; Maternal and infant health | Home | Information, education or awareness | Dose or dosage |
| [92] | Pawar, et al. | 2015 | India | Smoking | School | Information, education or awareness | Dose or dosage |
| [93] | Pearson, et al. | 2010 | UK | Diet and nutrition | Home; Community | Information, education or awareness | Dose or dosage |
| [94] | Pfeiffer, et al. | 2013 | US | Physical activity | School | Behavioural | Dose delivered |
| [95] | Plotnikoff, et al. | 2010 | Canada | Diet and nutrition; Physical activity | Workplace | Information, education or awareness | Dose or dosage |
| [96] | Poelman, et al. | 2013 | Netherlands | Diet and nutrition | Online | Information, education or awareness | Other: Exposure dose |
| [97] | Potter, et al. | 2008 | US | Physical activity | Community | Information, education or awareness | Dose-response |
| [98] | Raj, et al. | 2001 | US | Sexual health | Healthcare organisation; Recreation or community venue | Information, education or awareness | Dose or dosage |
| [99] | Reynolds, et al. | 2000 | US | Diet and nutrition | School | Information, education or awareness | Dose or dosage |
| [100] | Rijsdijk, et al. | 2014 | Uganda | Sexual health | School | Environmental | Dose delivered |
| [101] | Risica, et al. | 2013 | US | Weight management; Diet and nutrition; Physical activity | Home | Information, education or awareness | Dose or dosage |
| [102] | Robbins, et al. | 2014 | US | Weight management; Physical activity | School | Behavioural | Dose delivered; Dose received |
| [103] | Robert, et al. | 2007 | Peru | Maternal and infant health | Healthcare organisation | Information, education or awareness; Behavioural | Dose delivered |
| [104] | Rosecrans, et al. | 2008 | Canada | Diet and nutrition; Physical activity; Diabetes | School; Healthcare organisation; Food store | Information, education or awareness; Environmental | Dose delivered; Dose received |
| [105] | Ross, et al. | 2006 | Nigeria | Sexual health | Workplace | Capacity building; Behavioural | Dose-response |
| [106] | Rubinstein, et al. | 2016 | Argentina, Guatemala, and Peru | Weight management; Diet and nutrition; Physical activity; Cardiovascular disease | Online | Information, education or awareness; Behavioural | Dose or dosage |
| [107] | Sanchez, et al. | 2014 | Mexico | Cardiovascular disease | Healthcare organisation; Community | Information, education or awareness | Dose or dosage |
| [108] | Sharma, et al. | 2015 | US | Diet and nutrition; Physical activity | School | Information, education or awareness | Dose or dosage; Dose-response |
| [109] | Sherwood, et al. | 2010 | US | Diet and nutrition | Home | Information, education or awareness; Behavioural | Dose or dosage |
| [110] | Smith, et al. | 2014 | Australia | Weight management; Diet and nutrition; Physical activity | School | Information, education or awareness; Behavioural | Dose or dosage |
| [111] | Stone, et al. | 2003 | US | Weight management; Diet and nutrition; Physical activity | School | Information, education or awareness; Environmental; Behavioural | Dose or dosage |
| [112] | Story, et al. | 2000 | US | Diet and nutrition | School | Information, education or awareness; Environmental; Behavioural | Dose or dosage |
| [113] | Strijk, et al. | 2011 | Netherlands | Wellbeing and mental health | Workplace | Behavioural | Dose delivered; Dose received |
| [114] | Sy & Glanz | 2008 | US | Smoking | School |  | Dose or dosage |
| [115] | Thompson, et al. | 2008 | US | Weight management; Diet and nutrition; Physical activity | Online | Behavioural | Dose or dosage |
| [116] | Thomson, et al. | 2015 | US | Diet and nutrition; Physical activity | Faith organisation | Information, education or awareness; Behavioural | Other: Dose intensity |
| [117] | Tolma, et al. | 2009 | US | Other: Community health partnership | Community | Capacity building | Dose delivered; Dose received |
| [118] | Trigwell, et al. | 2015 | UK | Smoking | School | Behavioural | Dose or dosage |
| [119] | van Schijndel-Speet, et al. | 2014 | Netherlands | Physical activity | Recreation or community venue | Behavioural | Dose delivered; Dose received |
| [120] | Walkosz, et al. | 2008 | US and Canada | Cancer | Community | Information, education or awareness | Dose or dosage |
| [121] | Wang, et al. | 2013 | US | Diet and nutrition; Physical activity; Diabetes | Faith organisation; Community | Information, education or awareness; Environmental | Dose delivered; Dose received |
| [122] | Waqa, et al. | 2013 | Fiji | Weight management; Diet and nutrition; Physical activity | School; Faith organisation | Capacity building | Dose-response |
| [123] | Wierenga, et al. | 2014 | Netherlands | Diet and nutrition; Smoking; Physical activity; Wellbeing and mental health | Workplace | Behavioural | Dose delivered; Dose received |
| [124] | Williamson, et al. | 2001 | UK | Sexual health | Recreation or community venue | Information, education or awareness | Dose or dosage |
| [125] | Wilson, et al. | 2010 | US | Physical activity | Workplace | Environmental; Behavioural | Dose delivered; Dose received |
| [126] | Wolfers, et al. | 2011 | Netherlands | Sexual health | School | Information, education or awareness | Dose or dosage |
| [127] | Yap & Busch James | 2010 | US | Physical activity | Workplace | Information, education or awareness | Dose or dosage |
| [128] | Yap, et al. | 2009 | US | Physical activity | Workplace | Information, education or awareness | Dose or dosage |
| [129] | Yin, et al. | 2005 | US | Weight management; Physical activity | School | Behavioural | Dose or dosage |
| [130] | Zoellner, et al. | 2011 | US | Physical activity | Community | Information, education or awareness; Behavioural | Other: Dose effects |

1. Aarestrup AK, Jorgensen TS, Jorgensen SE, Hoelscher DM, Due P, Krolner R. Implementation of strategies to increase adolescents' access to fruit and vegetables at school: process evaluation findings from the Boost study. BMC Public Health 2015;15.

2. Alia KA, Wilson DK, McDaniel T, St George SM, Kitzman-Ulrich H, Smith K et al. Development of an innovative process evaluation approach for the Families Improving Together (FIT) for weight loss trial in African American adolescents. Eval Program Plann 2015;49:106-16.

3. Androutsos O, Apostolidou E, Iotova V, Socha P, Birnbaum J, Moreno L et al. Process evaluation design and tools used in a kindergarten-based, family-involved intervention to prevent obesity in early childhood. The ToyBox-study. Obes Rev 2014;15:74-80.

4. Ayala GX. Effects of a promotor-based intervention to promote physical activity: familias sanas y activas. Am J Public Health 2011;101:2261-2268.

5. Baquero B, Linnan L, Laraia BA, Ayala GX. Process evaluation of a food marketing and environmental change intervention in Tiendas that serve Latino immigrants in North Carolina. Health Promot Pract 2014;15:839-48.

6. Baquero B, Ayala GX, Arredondo EM, Campbell NR, Slymen DJ, Gallo L et al. Secretos de la Buena Vida: processes of dietary change via a tailored nutrition communication intervention for Latinas. Health Educ Res 2009;24:855-866.

7. Baranowski T, Baranowski JC, Cullen KW, Thompson DI, Nicklas T, Zakeri IE et al. The Fun, Food, and Fitness Project (FFFP): the Baylor GEMS pilot study. Ethn Dis 2003;13:S30-9.

8. Bellows LL, Davies PL, Anderson J, Kennedy C. Effectiveness of a physical activity intervention for Head Start preschoolers: a randomized intervention study. Effectiveness of a physical activity intervention for Head Start preschoolers: a randomized intervention study 2013;67:28-36.

9. Berendsen BAJ, Kremers SPJ, Savelberg HHCM, Schaper NC, Hendriks MRC. The implementation and sustainability of a combined lifestyle intervention in primary care: mixed method process evaluation. BMC Fam Pract 2015;16:37.

10. Beresford SA, Shannon J, McLerran D, Thompson B. Seattle 5-a-Day Work-Site Project: process evaluation. Health Educ Behav 2000;27:213-22.

11. Berkowitz JM, Huhman M, Nolin MJ. Did augmenting the VERBTM campaign advertising in select communities have an effect on awareness, attitudes, and physical activity? Am J Prev Med 2008;34:S257-S266.

12. Binkley CJ, Johnson KW, Abadi M, Thompson K, Shamblen SR, Young L et al. Improving the oral health of residents with intellectual and developmental disabilities: an oral health strategy and pilot study. Eval Program Plann 2014;47:54-63.

13. Birnbaum AS, Lytle LA, Story M, Perry CL, Murray DM. Are differences in exposure to a multicomponent school-based intervention associated with varying dietary outcomes in adolescents? Health Educ Behav 2002;29:427-43.

14. Bishop DC, Dusenbury L, Pankratz MM, Hansen WB. Promoting quality of program delivery via an internet message delivery system. J Drug Educ 2013;43:235-54.

15. Bjelland M, Bergh IH, Grydeland M, Klepp K-I, Andersen LF, Anderssen SA et al. Changes in adolescents' intake of sugar-sweetened beverages and sedentary behaviour: results at 8 month mid-way assessment of the HEIA study--a comprehensive, multi-component school-based randomized trial. Int J Behav Nutr Phys Act 2011;8:63.

16. Bolier L, Haverman M, Kramer J, Westerhof GJ, Riper H, Walburg JA et al. An Internet-based intervention to promote mental fitness for mildly depressed adults: randomized controlled trial. J Med Internet Res 2013;15:209-226.

17. Bowen AM, Williams ML, Daniel CM, Clayton S. Internet based HIV prevention research targeting rural MSM: feasibility, acceptability, and preliminary efficacy. J Behav Med 2008;31:463-77.

18. Branscum P, Sharma M, Wang LL, Wilson B, Rojas-Guyler L. A process evaluation of a social cognitive theory-based childhood obesity prevention intervention: the comics for health program. Health Promot Pract 2013;14:189-198.

19. Broekhuizen K, Althuizen E, van Poppel MNM, Donker M, van Mechelen W. From theory to practice: Intervention fidelity in a randomized controlled trial aiming to optimize weight development during pregnancy. Health Promot Pract 2012;13:816-825.

20. Buckley L, Sheehan M. A process evaluation of an injury prevention school-based programme for adolescents. Health Educ Res 2009;24:507-19.

21. Buller DB, Andersen PA, Walkosz BJ, Scott MD, Cutter GR, Dignan MB et al. Randomized Trial Testing a Worksite Sun Protection Program in an Outdoor Recreation Industry. Health Educ Behav 2005;32:514-535.

22. Buller DB, Borland R, Woodall WG, Hall JR, Hines JM, Burris-Woodall P et al. Randomized trials on consider this, a tailored, internet-delivered smoking prevention program for adolescents. Health Educ Behav 2008;35:260-81.

23. Caldera D, Burrell L, Rodriguez K, Crowne SS, Rohde C, Duggan A. Impact of a statewide home visiting program on parenting and on child health and development. Child Abuse Negl 2007;31:829-52.

24. Campbell MK, Resnicow K, Carr C, Wang T, Williams A. Process evaluation of an effective church-based diet intervention: Body & Soul. Health Educ Behav 2007;34:864-880.

25. Campbell MK, Carbone E, Honess-Morreale L, Heisler-Mackinnon J, Demissie S, Farrell D. Randomized trial of a tailored nutrition education CD-ROM program for women receiving food assistance. J Nutr Educ Behav 2004;36:58-66.

26. Cargo M, Salsberg J, Delormier T, Desrosiers S, Macaulay AC. Understanding the social context of school health promotion program implementation. Health Education 2006;106:85-97.

27. Cheadle A, Schwartz P, Rauzon S. CA4-04: Using the Concept of “Population Dose” in Planning and Evaluating Community Health Initiatives. In: *Health Maintenance Organization Research Network.* vol. 10. Seattle, Washington: Marshfield Clinic; 2012: 184-185.

28. Chen X, Ren Y, Lin F, MacDonell K, Jiang Y. Exposure to school and community based prevention programs and reductions in cigarette smoking among adolescents in the United States, 2000-08. Eval Program Plann 2012;35:321-8.

29. Chittleborough CR, Nicholson AL, Young E, Bell S, Campbell R. Implementation of an educational intervention to improve hand washing in primary schools: process evaluation within a randomised controlled trial. BMC Public Health 2013;13:757.

30. Coffeng JK, Hendriksen IJM, van Mechelen W, Boot CRL. Process evaluation of a worksite social and physical environmental intervention. ‎J Occup Environ Med 2013;55:1409-20.

31. Cook RF, Billings DW, Hersch RK, Back AS, Hendrickson A. A field test of a web-based workplace health promotion program to improve dietary practices, reduce stress, and increase physical activity: randomized controlled trial. J Med Internet Res 2007;9:e17.

32. Cook RF, Hersch RK, Schlossberg D, Leaf SL. A Web-based health promotion program for older workers: randomized controlled trial. J Med Internet Res 2015;17:e82.

33. Coulon SM, Wilson DK, Griffin S, St George SM, Alia KA, Trumpeter NN et al. Formative process evaluation for implementing a social marketing intervention to increase walking among African Americans in the Positive Action for Today's Health trial. Formative process evaluation for implementing a social marketing intervention to increase walking among African Americans in the Positive Action for Today's Health trial 2012;102:2315-21.

34. Cowan JA, Devine CM. Process evaluation of an environmental and educational nutrition intervention in residential drug-treatment facilities. Public Health Nutr 2012;15:1159-67.

35. Crutzen R, Roosjen JL, Poelman J. Using Google Analytics as a process evaluation method for Internet-delivered interventions: an example on sexual health. Health Promot Int 2013;28:36-42.

36. Curran S, Gittelsohn J, Anliker J, Ethelbah B, Blake K, Sharma S et al. Process evaluation of a store-based environmental obesity intervention on two American Indian Reservations. Health Educ Res 2005;20:719-29.

37. Day ME, Strange KS, McKay HA, Naylor P-J. Action schools! BC--Healthy Eating: effects of a whole-school model to modifying eating behaviours of elementary school children. Can J Public Health 2008;99:328-31.

38. Devine CM, Maley M, Farrell TJ, Warren B, Sadigov S, Carroll J. Process evaluation of an environmental walking and healthy eating pilot in small rural worksites. Eval Program Plann 2012;35:88-96.

39. DiClemente RJ, Wingood GM, Sales JM, Brown JL, Rose ES, Davis TL et al. Efficacy of a telephone-delivered sexually transmitted infection/human immunodeficiency virus prevention maintenance intervention for adolescents: a randomized clinical trial. JAMA Pediatr 2014;168:938-46.

40. Fagan AA, Hanson K, Hawkins JD, Arthur MW. Bridging science to practice: achieving prevention program implementation fidelity in the community youth development study. Am J Community Psychol 2008;41:235-49.

41. Farrelly MC, Davis KC, Haviland ML, Messeri P, Healton CG. Evidence of a dose-response relationship between "truth" antismoking ads and youth smoking prevalence. Am J Public Health 2005;95:425-31.

42. Fort MP, Murillo S, Lopez E, Dengo AL, Alvarado-Molina N, Beausset Id et al. Impact evaluation of a healthy lifestyle intervention to reduce cardiovascular disease risk in health centers in San Jose, Costa Rica and Chiapas, Mexico. Impact evaluation of a healthy lifestyle intervention to reduce cardiovascular disease risk in health centers in San Jose, Costa Rica and Chiapas, Mexico 2015;15.

43. Fotu KF, Moodie MM, Mavoa HM, Pomana S, Schultz JT, Swinburn BA. Process evaluation of a community-based adolescent obesity prevention project in Tonga. BMC Public Health 2011;11:284.

44. Freedman DA, Choi SK, Hurley T, Anadu E, Hebert JR. A farmers' market at a federally qualified health center improves fruit and vegetable intake among low-income diabetics. Prev Med 2013;56:288-292.

45. Gittelsohn J, Dennisuk LA, Christiansen K, Bhimani R, Johnson A, Alexander E et al. Development and implementation of Baltimore Healthy Eating Zones: a youth-targeted intervention to improve the urban food environment. Health Educ Res 2013;28:732-744.

46. Gittelsohn J, Suratkar S, Song HJ, Sacher S, Rajan R, Rasooly IR et al. Process evaluation of Baltimore Healthy Stores: a pilot health intervention program with supermarkets and corner stores in Baltimore City. Health Promot Pract 2010;11:723-732.

47. Goenka S, Tewari A, Arora M, Stigler MH, Perry CL, Arnold JPS et al. Process evaluation of a tobacco prevention program in Indian schools--methods, results and lessons learnt. Health Educ Res 2010;25:917-35.

48. Goode AD, Winkler EAH, Lawler SP, Reeves MM, Owen N, Eakin EG. A telephone-delivered physical activity and dietary intervention for type 2 diabetes and hypertension: does intervention dose influence outcomes? Am J Health Promot 2011;25:257-263.

49. Griffin SF, Wilcox S, Ory MG, Lattimore D, Leviton L, Castro C et al. Results from the Active for Life process evaluation: program delivery fidelity and adaptations. Health Educ Res 2010;25:325-342.

50. Hall WJ, Zeveloff A, Steckler A, Schneider M, Thompson D, Trang P et al. Process evaluation results from the HEALTHY physical education intervention. Health Educ Res 2012;27:307-318.

51. Havas S, Anliker J, Greenberg D, Block G, Block T, Blik C et al. Final results of the Maryland WIC Food for Life Program. Prev Med 2003;37:406-16.

52. Heelan KA, Bartee RT, Nihiser A, Sherry B. Healthier school environment leads to decreases in childhood obesity: the Kearney Nebraska story. Child Obes 2015;11:600-607.

53. Hillemeier MM, Downs DS, Feinberg ME, Weisman CS, Chuang CH, Parrott R et al. Improving women's preconceptional health: findings from a randomized trial of the Strong Healthy Women intervention in the Central Pennsylvania women's health study. Womens Health Issues 2008;18:S87-96.

54. Hoekstra F, Alingh RA, van der Schans CP, Hettinga FJ, Duijf M, Dekker R et al. Design of a process evaluation of the implementation of a physical activity and sports stimulation programme in Dutch rehabilitation setting: ReSpAct. Implement Sci 2014;9:127.

55. Huhman ME, Potter LD, Duke JC, Judkins DR, Heitzler CD, Wong FL. Evaluation of a national physical activity intervention for children: VERB campaign, 2002-2004. Am J Prev Med 2007;32:38-43.

56. Huhman ME, Potter LD, Nolin MJ, Piesse A, Judkins DR, Banspach SW et al. The Influence of the VERB campaign on children's physical activity in 2002 to 2006. The Influence of the VERB campaign on children's physical activity in 2002 to 2006 2010;100:638-45.

57. Hunt MK, Lederman R, Stoddard A, Potter S, Phillips J, Sorensen G. Process tracking results from the Treatwell 5-a-Day Worksite Study. Process tracking results from the Treatwell 5-a-Day Worksite Study 2000;14:179-87.

58. Hwang KO, Ottenbacher AJ, Graham AL, Thomas EJ, Street RL, Jr., Vernon SW. Online narratives and peer support for colorectal cancer screening: a pilot randomized trial. Am J Prev Med 2013;45:98-107.

59. Johnson CC, Lai Y, Rice J, Rose D, Webber LS. ACTION live: using process evaluation to describe implementation of a worksite wellness program. J Occup Environ Med 2010;52:S14-S21.

60. Kaphingst KA, Lobb R, Fay ME, Hunt MK, Suarez EG, Fletcher RH et al. Impact of intervention dose on cancer-related health behaviors among working-class, multiethnic, community health center patients. Impact of intervention dose on cancer-related health behaviors among working-class, multiethnic, community health center patients 2007;21:262-6.

61. Kaufman MR, Mooney A, Kamala B, Modarres N, Karam R, Ng'wanansabi D. Effects of the Fataki campaign: addressing cross-generational sex in Tanzania by mobilizing communities to intervene. Effects of the Fataki campaign: addressing cross-generational sex in Tanzania by mobilizing communities to intervene 2013;17:2053-62.

62. Keating SE, Hackett DA, Parker HM, O'Connor HT, Gerofi JA, Sainsbury A et al. Effect of aerobic exercise training dose on liver fat and visceral adiposity. J Hepatol 2015;63:174-182.

63. Kelishadi R, Sarrafzadegan N, Sadri GH, Pashmi R, Mohammadifard N, Tavasoli AA et al. Short-term results of a community-based program on promoting healthy lifestyle for prevention and control of chronic diseases in a developing country setting: Isfahan Healthy Heart Program. Asia Pac J Public Health 2011;23:518-33.

64. Kim SS, Ali D, Kennedy A, Tesfaye R, Tadesse AW, Abrha TH et al. Assessing implementation fidelity of a community-based infant and young child feeding intervention in Ethiopia identifies delivery challenges that limit reach to communities: a mixed-method process evaluation study. BMC Public Health 2015;15:316.

65. Knowlden AP, Sharma M. Process evaluation of the Enabling Mothers to Prevent Pediatric Obesity Through Web-Based Learning and Reciprocal Determinism (EMPOWER) randomized control trial. Health Promot Pract 2014;15:685-694.

66. Koniak-Griffin D, Brecht M-L, Takayanagi S, Villegas J, Melendrez M, Balcazar H. A community health worker-led lifestyle behavior intervention for Latina (Hispanic) women: feasibility and outcomes of a randomized controlled trial. Int J Nurs Stud 2015;52:75-87.

67. Kozica SL, Lombard CB, Ilic D, Ng S, Harrison CL, Teede HJ. Acceptability of delivery modes for lifestyle advice in a large scale randomised controlled obesity prevention trial. BMC Public Health 2015;15:699.

68. Kulwa Kb VRBKPMPSKPWLC. Effectiveness of a nutrition education package in improving feeding practices, dietary adequacy and growth of infants and young children in rural Tanzania: rationale, design and methods of a cluster randomised trial. BMC Public Health 2014;14:1077.

69. Lane A, Murphy N, Bauman A. An effort to 'leverage' the effect of participation in a mass event on physical activity. Health Promot Int 2015;30:542-551.

70. Lee JGL, Ranney LM, Goldstein AO. Cigarette butts near building entrances: what is the impact of smoke-free college campus policies? Tob Control 2013;22:107-112.

71. Lee RM, Rothstein JD, Gergen J, Zachary DA, Smith JC, Palmer AM et al. Process evaluation of a comprehensive supermarket intervention in a low-income Baltimore community. Health Promot Pract 2015;16:849-858.

72. Lee-Kwan SH, Goedkoop S, Yong R, Batorsky B, Hoffman V, Jeffries J et al. Development and implementation of the Baltimore healthy carry-outs feasibility trial: process evaluation results. BMC Public Health 2013;13.

73. Lemon SC, Zapka J, Li W, Estabrook B, Rosal M, Magner R et al. Step ahead: a worksite obesity prevention trial among hospital employees. Am J Prev Med 2010;38:27-38.

74. Magnani R, MacIntyre K, Karim AM, Brown L, Hutchinson P. The impact of life skills education on adolescent sexual risk behaviors in KwaZulu-Natal, South Africa. J Adolesc Health 2005;36:289-304.

75. Marcus BH, Hartman SJ, Pekmezi D, Dunsiger SI, Linke SE, Marquez B et al. Using interactive Internet technology to promote physical activity in Latinas: rationale, design, and baseline findings of Pasos Hacia La Salud. Contemp Clin Trials 2015;44:149-158.

76. Mathews LB, Moodie MM, Simmons AM, Swinburn BA. The process evaluation of It's Your Move!, an Australian adolescent community-based obesity prevention project. BMC Public Health 2010;10.

77. McAlister A, Morrison TC, Hu S, Meshack AF, Ramirez A, Gallion K et al. Media and community campaign effects on adult tobacco use in Texas. J Health Commun 2004;9:95-109.

78. McCreary LL, Kaponda CPN, Kafulafula UK, Ngalande RC, Kumbani LC, Jere DLN et al. Process evaluation of HIV prevention peer groups in Malawi: a look inside the black box. Health Educ Res 2010;25:965-78.

79. Mitchell DC, Andrews T, Schenker MB. Pasos saludables: A pilot randomized intervention study to reduce obesity in an immigrant farmworker population. ‎J Occup Environ Med 2015;57:1039-1046.

80. Murrock CJ, Gary FA. Culturally specific dance to reduce obesity in African American women. Health Promot Pract 2010;11:465-473.

81. Myers CA, Johnson WD, Earnest CP, Rood JC, Tudor-Locke C, Johannsen NM et al. Examination of mechanisms (E-MECHANIC) of exercise-induced weight compensation: study protocol for a randomized controlled trial. Trials 2014;15:212.

82. Nassau Fv, Singh AS, Mechelen Wv, Paulussen TGWM, Brug J, Chinapaw MJM. Exploring facilitating factors and barriers to the nationwide dissemination of a Dutch school-based obesity prevention program "DOiT-": a study protocol. BMC Public Health 2013;13.

83. Nathan S, Bunde-Birouste A, Evers C, Kemp L, MacKenzie J, Henley R. Social cohesion through football: a quasi-experimental mixed methods design to evaluate a complex health promotion program. BMC Public Health 2010;10.

84. Nicklas TA, O'Neil CE. Process of conducting a 5-a-day intervention with high school students: Gimme 5 (Louisiana). Health Educ Behav 2000;27:201-12.

85. Nicklas TA, Nguyen T, Butte NF, Liu Y. The children in action pilot study. Int J Child Health Nutr 2013;2:296-308.

86. Nicklas TA, Goh ET, Goodell LS, Acuff DS, Reiher R, Buday R et al. Impact of commercials on food preferences of low-income, minority preschoolers. J Nutr Educ Behav 2011;43:35-41.

87. O'Hara BJ, Bauman AE, King EL, Phongsavan P. Process evaluation of the advertising campaign for the NSW Get Healthy Information and Coaching Service. Health Promot J Austr 2011;22:68-71.

88. Ostwald SK, Godwin KM, Cron SG, Kelley CP, Hersch G, Davis S. Home-based psychoeducational and mailed information programs for stroke-caregiving dyads post-discharge: a randomized trial. Disabil Rehabil 2014;36:55-62.

89. Oude Hengel KM, Blatter BM, van der Molen HF, Joling CI, Proper KI, Bongers PM et al. Meeting the challenges of implementing an intervention to promote work ability and health-related quality of life at construction worksites: a process evaluation. ‎J Occup Environ Med 2011;53:1483-91.

90. Parker KB, DeJoy DM, Wilson MG, Bowen HM, Goetzel RZ. Application of the Environmental Assessment Tool (EAT) as a process measure for a worksite weight management intervention. J Occup Environ Med. 2010;52:S42-S51.

91. Paul KH, Olson CM. Moving beyond quantity of participation in process evaluation of an intervention to prevent excessive pregnancy weight gain. Int J Behav Nutr Phys Act 2013;10.

92. Pawar PS, Nagler EM, Gupta PC, Stoddard AM, Lando HA, Shulman L et al. Tracking intervention delivery in the 'Tobacco-Free Teachers/Tobacco-Free Society' program, Bihar, India. Health Educ Res 2015;30:731-741.

93. Pearson N, Atkin AJ, Biddle SJH, Gorely T. A family-based intervention to increase fruit and vegetable consumption in adolescents: a pilot study. Public Health Nutr 2010;13:876-85.

94. Pfeiffer KA, Saunders RP, Brown WH, Dowda M, Addy CL, Pate RR. Study of Health and Activity in Preschool Environments (SHAPES): study protocol for a randomized trial evaluating a multi-component physical activity intervention in preschool children. BMC Public Health 2013;13.

95. Plotnikoff RC, Pickering MA, McCargar LJ, Loucaides CA, Hugo K. Six-month follow-up and participant use and satisfaction of an electronic mail intervention promoting physical activity and nutrition. Am J Health Promot 2010;24:255-259.

96. Poelman MP, Steenhuis IHM, de Vet E, Seidell JC. The development and evaluation of an Internet-based intervention to increase awareness about food portion sizes: a randomized, controlled trial. J Nutr Educ Behav 2013;45:701-7.

97. Potter LD, Judkins DR, Piesse A, Nolin MJ, Huhman M. Methodology of the outcome evaluation of the VERB campaign. Methodology of the outcome evaluation of the VERB campaign 2008;34:S230-40.

98. Raj A, Amaro H, Cranston K, Martin B, Cabral H, Navarro A et al. Is a general women's health promotion program as effective as an HIV-intensive prevention program in reducing HIV risk among Hispanic women? Public Health Rep 2001;116:599-607.

99. Reynolds KD, Franklin FA, Leviton LC, Maloy J, Harrington KF, Yaroch AL et al. Methods, results, and lessons learned from process evaluation of the high 5 school-based nutrition intervention. Health Educ Behav 2000;27:177-86.

100. Rijsdijk LE, Bos AER, Lie R, Leerlooijer JN, Eiling E, Atema V et al. Implementation of The World Starts With Me, a comprehensive rights-based sex education programme in Uganda. Health Educ Res 2014;29:340-353.

101. Risica PM, Gans KM, Kumanyika S, Kirtania U, Lasater TM. SisterTalk: final results of a culturally tailored cable television delivered weight control program for Black women. Int J Behav Nutr Phys Act 2013;10.

102. Robbins LB, Pfeiffer KA, Wesolek SM, Lo Y-J. Process evaluation for a school-based physical activity intervention for 6th- and 7th-grade boys: reach, dose, and fidelity. Eval Program Plann 2014;42:21-31.

103. Robert RC, Gittelsohn J, Creed-Kanashiro HM, Penny ME, Caulfield LE, Narro MR et al. Implementation examined in a health center-delivered, educational intervention that improved infant growth in Trujillo, Peru: successes and challenges. Health Educ Res 2007;22:318-331.

104. Rosecrans AM, Gittelsohn J, Ho LS, Harris SB, Naqshbandi M, Sharma S. Process evaluation of a multi-institutional community-based program for diabetes prevention among First Nations. Health Educ Res 2008;23:272-86.

105. Ross MW, Essien EJ, Ekong E, James TM, Amos C, Ogungbade GO et al. The impact of a situationally focused individual human immunodeficiency virus/sexually transmitted disease risk-reduction intervention on risk behavior in a 1-year cohort of Nigerian military personnel. Mil Med 2006;171:970-5.

106. Rubinstein A, Miranda JJ, Beratarrechea A, Diez-Canseco F, Kanter R, Gutierrez L et al. Effectiveness of an mHealth intervention to improve the cardiometabolic profile of people with prehypertension in low-resource urban settings in Latin America: a randomised controlled trial. Lancet Diabetes Endocrinol 2016;4:52-63.

107. Sanchez V, Cacari Stone L, Moffett ML, Nguyen P, Muhammad M, Bruna-Lewis S et al. Process evaluation of a promotora de salud intervention for improving hypertension outcomes for Latinos living in a rural U.S.-Mexico border region. Health Promot Pract 2014;15:356-64.

108. Sharma SV, Shegog R, Chow J, Finley C, Pomeroy M, Smith C et al. Effects of the Quest to Lava Mountain computer game on dietary and physical activity behaviors of elementary school children: a pilot group-randomized controlled trial. J Acad Nutr Diet 2015;115:1260-1271.

109. Sherwood NE, Jeffery RW, Welsh EM, VanWormer J, Hotop AM. The drop it at last study: six-month results of a phone-based weight loss trial. Am J Health Promot 2010;24:378-383.

110. Smith JJ, Morgan PJ, Plotnikoff RC, Dally KA, Salmon J, Okely AD et al. Smart-phone obesity prevention trial for adolescent boys in low-income communities: the ATLAS RCT. Pediatrics 2014;134:e723-e731.

111. Stone EJ, Norman JE, Davis SM, Stewart D, Clay TE, Caballero B et al. Design, implementation, and quality control in the Pathways American-Indian multicenter trial. Prev Med 2003;37:s13-s23.

112. Story M, Mays RW, Bishop DB, Perry CL, Taylor G, Smyth M et al. 5-a-day Power Plus: process evaluation of a multicomponent elementary school program to increase fruit and vegetable consumption. Health Educ Behav 2000;27:187-200.

113. Strijk JE, Proper KI, Beek AJvd, Mechelen Wv. A process evaluation of a worksite vitality intervention among ageing hospital workers. Int J Behav Nutr Phys Act 2011;8.

114. Sy A, Glanz K. Factors influencing teachers' implementation of an innovative tobacco prevention curriculum for multiethnic youth: Project SPLASH. J School Health 2008;78:264-273.

115. Thompson D, Baranowski T, Cullen K, Watson K, Canada A, Bhatt R et al. Food, Fun and Fitness Internet program for girls: influencing log-on rate. Health Educ Res 2008;23:228-37.

116. Thomson JL, Goodman MH, Tussing-Humphreys L. Diet quality and physical activity outcome improvements resulting from a church-based diet and supervised physical activity intervention for rural, Southern, African American adults: Delta Body and Soul III. Health Promot Pract 2015;16:677-688.

117. Tolma EL, Cheney MK, Troup P, Hann N. Designing the process evaluation for the collaborative planning of a local turning point partnership. Health Promot Pract 2009;10:537-48.

118. Trigwell J, McGee CE, Murphy RC, Porcellato LA, Ussher M, Garnham-Lee K et al. Process evaluation of a sport-for-health intervention to prevent smoking amongst primary school children: SmokeFree Sports. BMC Public Health 2015;15:347.

119. van Schijndel-Speet M, Evenhuis HM, van Wijck R, Echteld MA. Implementation of a group-based physical activity programme for ageing adults with ID: a process evaluation. J Eval Clin Pract 2014;20:401-7.

120. Walkosz BJ, Buller DB, Andersen PA, Scott MD, Dignan MB, Cutter GR et al. Increasing sun protection in winter outdoor recreation: a theory-based health communication program. Increasing sun protection in winter outdoor recreation: a theory-based health communication program 2008;34:502-509.

121. Wang HE, Lee M, Hart A, Summers AC, Anderson Steeves E, Gittelsohn J. Process evaluation of Healthy Bodies, Healthy Souls: a church-based health intervention program in Baltimore City. Health Educ Res 2013;28:392-404.

122. Waqa G, Moodie M, Schultz J, Swinburn B. Process evaluation of a community-based intervention program: Healthy Youth Healthy Communities, an adolescent obesity prevention project in Fiji. Glob Health Promot 2013;20:23-34.

123. Wierenga D, Engbers LH, Van Empelen P, De Moes KJ, Wittink H, Grundemann R et al. The implementation of multiple lifestyle interventions in two organizations: a process evaluation. ‎J Occup Environ Med 2014;56:1195-206.

124. Williamson LM, Hart GJ, Flowers P, Frankis JS, Der GJ. The Gay Men's Task Force: the impact of peer education on the sexual health behaviour of homosexual men in Glasgow. Sex Transm Infect 2001;77:427-432.

125. Wilson MG, Basta TB, Bynum BH, DeJoy DM, Vandenberg RJ, Dishman RK. Do intervention fidelity and dose influence outcomes? Results from the Move to Improve worksite physical activity program. Health Educ Res 2010;25:294-305.

126. Wolfers M, Kok G, Looman C, de Zwart O, Mackenbach J. Promoting STI testing among senior vocational students in Rotterdam, the Netherlands: effects of a cluster randomized study. BMC Public Health 2011;11:937.

127. Yap TL, Busch James DM. Tailored e-mails in the workplace. Tailored e-mails in the workplace 2010;58:425-32.

128. Yap TL, Davis LS, Gates DM, Hemmings AB, Pan W. The effect of tailored E-mails in the workplace. Part I. Stage movement toward increased physical activity levels. The effect of tailored E-mails in the workplace. Part I. Stage movement toward increased physical activity levels 2009;57:267-73.

129. Yin Z, Moore JB, Johnson MH, Barbeau P, Cavnar M, Thornburg J et al. The Medical College of Georgia FitKid Project: the relations between program attendance and changes in outcomes in year 1. Int J Obes 2005;29:S40-S45.

130. Zoellner JM, Connell CC, Madson MB, Wang B, Reed VB, Molaison EF et al. H.U.B City Steps: methods and early findings from a community-based participatory research trial to reduce blood pressure among African Americans. Int J Behav Nutr Phys Act 2011;8.
